# AIB1 predicts tumor response to definitive chemoradiotherapy and prognosis in cervical squamous cell carcinoma

**DOI:** 10.7150/jca.31697

**Published:** 2019-08-28

**Authors:** Zhenfeng Zhao, Shuguang Zhou, Wenyu Li, Fei Zhong, Heping Zhang, Lei Sheng, Yue Li, Meng Xu, Jifei Xu, Lei Zhan, Bao Li, Fan Wang, Dan Xie, Zhuting Tong

**Affiliations:** 1 Department of Radiation Oncology, The First Affiliated Hospital of Anhui Medical University, Hefei, China; 2 Department of Radiation Oncology, The Fourth Affiliated Hospital of Anhui Medical University, Hefei, China; 3 Department of Gynecology, Maternity and Child Healthcare Hospital of Anhui Medical University, Hefei, China; 4 Department of Oncology, Fuyang Hospital of Anhui Medical University, Fuyang, Anhui, China; 5 Department of Pathology, Maternity and Child Healthcare Hospital of Anhui Medical University, Hefei, China; 6 Pathology Department of Anhui Medical University, Hefei, China; 7 The Comprehensive Lab, College of Basic medicine, Anhui Medical University, Hefei, China; 8 State Key Laboratory of Oncology in South China; Collaborative Innovation Center for Cancer Medicine; Department of Pathology, Sun Yat-sen University Cancer Center, Guangzhou, China

**Keywords:** Cervical cancer, AIB1, immunohistochemistry, chemoradiotherapy, Linear-Quadratic Model

## Abstract

Amplified in breast cancer 1 (AIB1) gene, has been reported to be associated with biological malignancy in several cancers. However, the molecular status of the AIB1 gene in cervical cancer and the clinicopathological/prognostic significance of AIB1 expression in chemoradiotherapy (CRT) sensitivity have not been determined. In our present study, we found that the high expression of AIB1 was frequent detected in specimens of cervical cancer patients, and this was significantly correlated with CRT response (*P* = 0.014), clinical stage (*P* = 0.003), T status (*P* = 0.027), N status (*P* = 0.021), M status (*P* = 0.015) and progression-free survival (*P* < 0.001). Moreover, the clonogenic survival fraction and cell apoptosis experiments showed that knockdown of AIB1 substantially increased cervical cancer cells sensitivity to ionizing radiation (IR) or cisplatin/5-fluorouracil. Collectively, our results demonstrated that the high expression of AIB1 in cervical cancer cells contributes to the resistance to CRT, which provides the evidence that AIB1 may be a promising predictor of aggressive cervical cancer patients with poor response to CRT.

## Introduction

Cervical cancer is one of the most common incident female cancers in the world, which accounts for the second cancer-related deaths in women, especially in developing countries 1, 2. The most common pathological type of cervical cancer is squamous cell carcinoma. In the developing countries, due to lack of healthcare awareness, majority of cervical cancer patients were diagnosed with advanced stage, and has lost the opportunity to perform radical operation 3, 4. Thus, the definitive chemoradiotherapy (CRT) become the standard treatment for locally advanced cervical cancer. Although advances in diagnosis, radiotherapy technology and management, the clinical prognosis of patients with advanced cervical cancer remains poor, due to eventual tumor recurrence and acquired chemoradiotherapy resistance. The 5‑year survival rate of advanced cervical cancer (III and IV stage) was reported less than 30% 5.

Currently, certain clinicopathological variables, including tumor size, regional lymph node status and parametrical infiltration, are widely accepted as prognostic factors. Additionally, several candidated genes, such as P53 6, Twist1 7, epidermal growth factor receptor (EGFR) 8, and vascular endothelial growth factor (VEGF) 9 had been identified as potential predictors of CRT sensitivity and/or prognosis of cervical cancer. However, all these factors and molecular markers are still insufficient to predict the prognosis and CRT response accurately. Thus, it is necessary to identify novel markers that can predict responses to CRT, so as to find novel therapeutic targets and develop individualized modalities of treatment for cervical cancer.

Amplified in breast cancer 1 (AIB1) gene, also known as ACTR, p/CIP, SRC3, RAC3, and TRAM1, is a member of the p160 family of steroid receptor coactivators 10-14. Initially, it has been identified as an oncogenic coactivator for nuclear steroid hormone receptors in hormone dependent cancers. There are cumulative evidences indicated that high expression of AIB1 contributes to tumor progression, metastasis, relapse and resistance of endocrine therapy in hormone-dependent tumor 15-19. Intriguingly, a discordant study reports that AIB1 overexpression is correlated with the absence of estrogen and progesterone receptors in breast cancer 20, suggesting that AIB1 may also function through hormone-independent pathway during tumorigenesis. Consistent with this finding, a majority of clinical studies showed that the upregulated expression of AIB1 was correlated with tumor aggressiveness and/or poor patient prognosis in a series of human hormone-independent malignancies, including gliomas 21, gastric 22, hepatocellular^23^, and colorectal carcinomas 24. These results were supported by extensive investigations showing that AIB1 can interact with a broad spectrum of non-hormonal transcription factors, such as AP-1, E2F-1, STAT6, and NFκB 25-28. Thus, many signal pathways, other than hormone dependent pathways, can be affected by AIB1 in hormone-insensitive malignancies, and dysregulation of these signaling subsequently facilitates tumorigenesis by promoting cell proliferation, survival and metastasis.

Our previous studies have shown that, AIB1 overexpression seems to indicate an aggressive phenotype of non-muscle-invasive bladder cancer with high risk of disease progression after initial treatment, and our further functional and mechanistic study revealed that AIB1 promotes bladder cancer cell proliferation and tumor growth by activating the AKT pathway and acting as coactivator of E2F1^29^. To date, however, the molecular status of the AIB1 gene in cervical cancer and the clinicopathological/prognostic significance of AIB1 expression in CRT sensitivity have not been determined. In this study, we report that AIB1 is frequently high-expressed in cervical cancer specimen, and the overexpression of AIB1 is closely associated with CRT resistance in cervical cancer patients. Furthermore, we investigated the possible effects of AIB1 on chemo/radio-sensitivity of cervical cancer cells by lentivirus-mediated knockdown assay.

## Material and Methods

### Patients and samples

In this study, 108 samples of cervical cancer tissues were collected from The First Affiliated Hospital of Anhui Medical University (Hefei, China) and Anhui Women and Child Health Care Hospital (Hefei, China) between March 2009 and December 2014. In addition, 30 samples of normal cervical tissues were used for controls. The histological types of all 108 cases cervical cancer were squamous cell carcinoma. Among the 108 cervical cancer patients, 37 cases were diagnosed with lymph node metastasis and 26 cases were diagnosed with distant metastasis. All of these patients were unable to perform surgery for physical reasons or late clinical stages. Tumor staging was determined according to the American Joint Committee on Cancer (AJCC, 2017) TNM standard, and tumor classification and histological types followed the World Health Organization (WHO) standards. The study was approved by the Institute Research Medical Ethics Committee of Anhui Medical University.

All of the cases received concurrent chemoradiotherapy by three-dimensional conformal radiotherapy or intensity-modulated radiotherapy (IMRT) with 6 MV X-ray: the clinical target volume (CTV) includes the parametrium, draining lymphatics, and upper vagina, the CTV are typically treated with a definitive dose of approximately 45 Gy (40-50 Gy), with 1.8-2Gy per fraction and five fractions weekly. The primary cervical tumor is then boosted by brachytherapy, with an additional 30 to 40 Gy to point A (in LDR equivalent dose), with 5-6 Gy per fraction 30. Concurrent chemotherapy regimen was with cisplatin (75 mg/m^2^) and 5-fluorouracil (500mg/d/m^2^ for 4 days). After concurrent chemoradiotherapy, patients were continued to undergo 1-6 rounds of adjuvant chemotherapy.

### Followed up

Patients were followed up every 3 months during the first year, and then every 6 months in the next two years, and finally annually thereafter. Diagnostic examinations consisted of abdominal ultrasonography, pelvic enhancement magnetic resonance imaging (MRI), chest x-ray, and other diagnostic procedures when necessary to detect recurrence and/or metastasis. Disease progression is defined as a case of tumor recurrence (local progression) and/or new distant metastasis (distant progression). The effect of CRT was evaluated clinically for primary lesions based on gynecological examination and pelvic enhancement magnetic resonance imaging after CRT according to the revised RECIST guideline (version 1.1) 31. Complete Response (CR): All target lesions are disappeared. Any pathological lymph nodes (target and/or non-target) must have reduction to < 10 mm in short axis. Partial Response (PR): The target lesions should decrease ≥ 30% in the sum of diameters, with the baseline sum diameters as reference. Progressive Disease (PD): The target lesions increase ≥ 20% in the sum of diameters, taking as reference the smallest sum (includes the baseline sum) on study. Additionally, the sum must also demonstrate an absolute increase of at least 5 mm. The appearance of new lesions (no matter one or more new lesions) is considered progression. Stable Disease (SD): Neither sufficient shrinkage to qualify for PR nor sufficient increase to qualify for PD, taking as reference the smallest sum diameters while on study. We divided these categories into two groups: CR and non-CR (PR/SD/PD).

### Immunohistochemical staining and quantification

Immunohistochemical studies were performed using a standard streptavidin-biotin-peroxidase complex method described previously 29. To evaluate the results of AIB1 IHC staining, the cervical tissues were scored by evaluating the percentage of positive cells with nuclear expression of AIB1 in each tissue section. As our study showed that the positive staining percentage of AIB1 in the normal cervical tissues was ranged from 0% to 10%, thus, the high-expression of AIB1 was scored when >10% of tumor cells were positively stained in the nuclei. Two independent observers blinded to clinicopathological information performed scoring.

### Cell line and cell culture

Three cervical squamous cell carcinoma cell lines, HCC 94 (HPV16- and 18-), SiHa (HPV16+) and CaSki (HPV16+ and 18+) cells, were used for the assays: The HCC-94 cell line was derived from the nude mice xenograft from a surgical specimen of human cervical squamous cell carcinoma. The SiHa cell line was derived from a Japanese cervical cancer patient pathological diagnosed with squamous cell carcinoma grade II. The CaSki cell line was derived from epidermoid carcinoma of the cervix metastatic to the small bowel mesentery. All of the three cell lines were cultured in Dulbecco's modified Eagle's medium (DMEM) supplemented with 10% fetal bovine serum (FBS) at 37˚C in a 5% CO2 atmosphere.

### Knocking down of aib1 by lentiviral short hairpin RNA (shRNA)

The vector pLLU2G was kindly provided by Professor Peng Xiang (Center for Stem Cell Biology and Tissue Engineering, Sun Yat-sen University). This vector is derived from pLL3.7 and shRNA expression elements and contains separate GFP as well as elements required for lentiviral packaging 32. The target sequences of AIB1 for constructing lentiviral short hairpin RNA is 5'-AGACTCCTTAGGACCGCTT-3'. Packaging of viruses was performed by transient transfection of 293FT cells with a transfer plasmid and three packaging vectors (pCMV-VSVG, pRSV-REV, and pMDLg/pRRE). Seventy-two hours after transfection, the lentiviral particles were collected and filtered (0.45 μM filters, Millipore), then concentrated by ultracentrifugation at 50000g for 2.5 h at 4°C. Subsequently, we infected the cervical cancer cell lines SiHa and CaSki with the lentivirus in a 24 well plate. Four days after infection, the knockdown efficiency of AIB1 was examined by Western blotting.

### Western blotting

Equal amounts of cell lysates were resolved by SDS-polyacrylamide gel electrophoresis (PAGE) and transferred to polyvinylidene difluoride (PVDF) membrane (Millipore, Bed-ford, MA, USA) followed by incubating with primary antibodies against AIB1 (BD Transduction Laboratories); cleaved-caspase 3 (Asp175), (Cell Signaling Technology, Boston. MA) cleaved-PARP (Asp214); and α-tubulin (BD Transduction Laboratories), respectively. The immunoreactive proteins were detected with enhanced chemiluminescence detection reagents (Thermo Pierce, Cramlington, UK) according to the manufacturer's instructions. Bicinchoninic acid (BCA) assay was preformed to determined protein concentrations.

### Annexin V-FITC / propidium iodide (PI) apoptosis detected by flow cytometry

To evaluate the apoptotic cells, the amount of apoptosis was measured by staining cells with both Annexin V-FITC and PI. The apoptosis assay was performed using the protocol according to manufacturer's instructions (Vazyme Biotech, China). Each sample was then analyzed by flow cytometry (BD Biosciences, San Jose, CA, USA).

### Clonogenic assays after radiation and Linear-Quadratic Model

The cells were plated in 60 mm dishes with increased numbers of cells for each dose group (500-8000 cell/dose). 24 hours after seeding, cells were subjected to irradiate (0, 2, 4, 6, 8, or 10 Gy) using 6 MV linear accelerator with photons (Varian Associates Inc., Palo Alto, California). The source surface distance (SSD) was 100 cm and the field size were 30×30 cm^2^. Then the cells were cultured in complete medium for a period of 14-21 days to allow colony growth. Cells were fixed with paraformaldehyde and stained with Giemsa (Invitrogen). The surviving colonies (>50 cells/colony) were manually counted. Data from X-irradiated cells were normalized to the control cells (defined as 100% colony forming ability). Plating efficiencies and survival fractions were calculated to plot cell survival curves and acquire survival parameters. All experiments were performed in triplicate and data were presented as means ± SD. The radiation survival curves were computer fitted by using the linear quadratic (LQ) model by GraphPad Prism 5.0 (GraphPad, San Diego, CA): as Survival Fraction (SF) = exp (-αD-βD²). The Sensitization Enhancement Ratio (SER) was calculated as follows: SER=SF2 of experimental cells/ SF2 of the parallel control cells. SF2 means the survival fraction at dose of 2Gy (SF2).

### Determination of half maximal inhibitory concentration, IC50

Briefly, cells were seeded in 96-well plates and cultured. Cell viability was determined by A Queous One Solution MTS kit (Promega) according to the manufacturer's instructions, and the absorption was read at 490 nm. Curves were computer fitted by using the GraphPad Prism 5.0 (Graph Pad Software Inc., San Diego, CA, USA) and IC50 was calculated. Data was presented as means ± standard deviation (SD) from three independent experiments.

### Statistical methods

Statistical analysis was performed with the SPSS software (SPSS Standard version 24.0, SPSS). The relationship between the expression of AIB1 and clinicopathologic of cervical cancer was detected by chi-square test. The strength of the relationship was evaluated with the Pearson's contingency coefficient. Progression-free survival (PFS) was analyzed with the Kaplan-Meier method and compared by the log rank test; PFS was defined as the time from diagnosis to tumor progression. Multivariate survival analysis was performed on all parameters that were found to be significant on univariate analysis using the Cox regression model. *P* values of <0.05 were considered significant.

## Results

### Patient characteristics

The clinicopathological characteristics of the 108 patients were shown in Table 1. In accordance with the 8th edition of the TNM classification of the American Joint Committee on Cancer (AJCC, 2017), 32 patients were classified into Stage II, 42 cases were Stage III and 34 cases were Stage IV. All the patients received the same regimen of concurrent CRT described above. At the evaluation time, the CR and non-CR were achieved in 46 and 62 cases, respectively. The 62 patients who did not achieved CR were consist of 39 cases PR, 11 cases NC and 12 cases PD.

### Expression of AIB1 in cervical cancer

**Figure 1:**
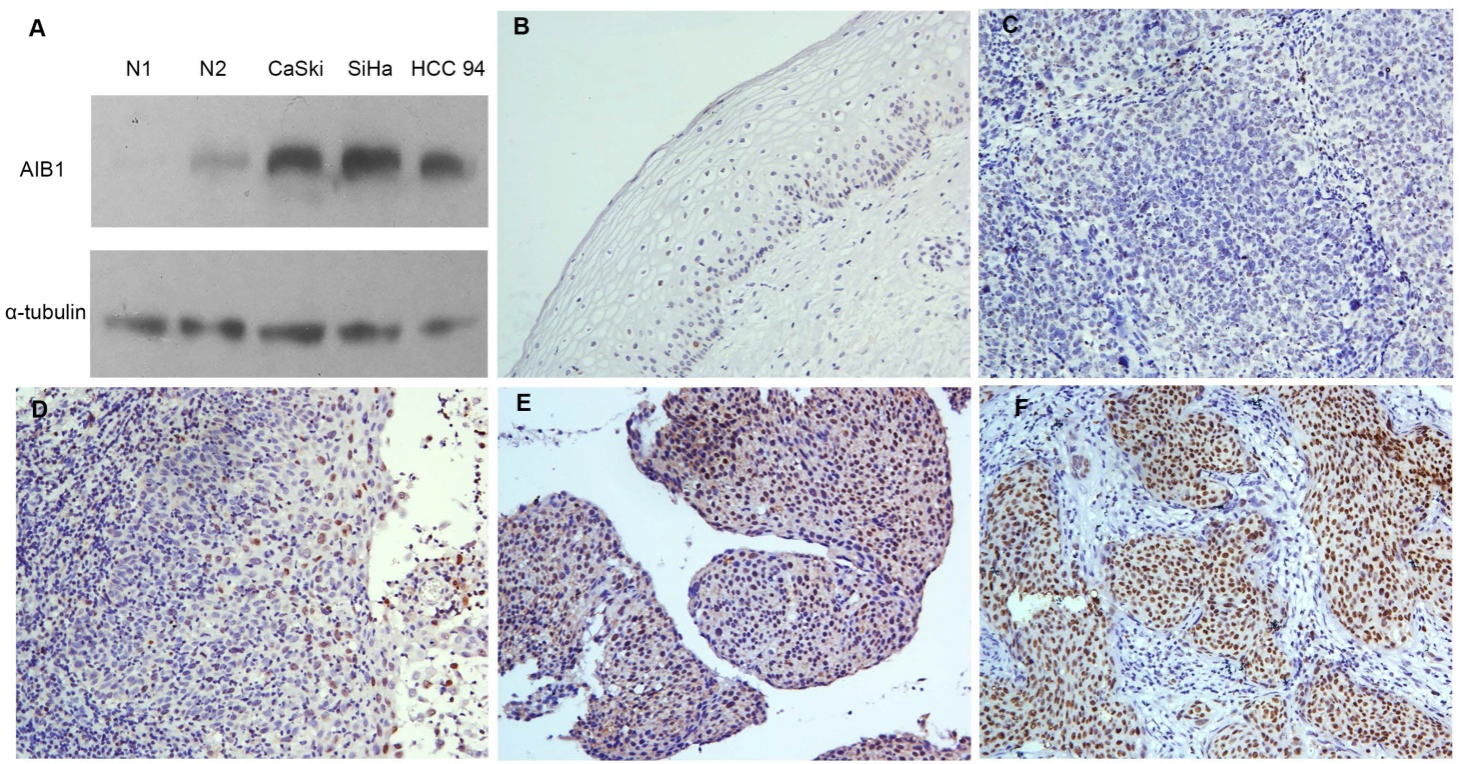
Expression of AIB1 in cervical cancer. **A*,*** The levels of AIB1 proteins in three cervical cancer cell lines and non-neoplastic cervical benign tissues examined by western blot. **B**, Normal cervical epithelial tissues (case 30) showed normal expression of AIB1 protein with a negative staining of AIB1 in the nuclei of all cervical epithelial cells (200×). **C**, Cervical squamous cell carcinoma (case 17) demonstrated normal expression of AIB1, in which all tumor cells showed negative staining of AIB1 (200×). **D**, Low expression of AIB1 was detected in cervical squamous cell carcinoma (case 34), in which less than 10% cancer cells showed low staining of AIB1 protein in the nuclei (200×). **E**, High expression of AIB1 was observed in cervical squamous cell carcinoma (case 21), in which 10~70% cancer cells demonstrated positive staining of AIB1 in the nuclei (200×). **F**, Another cervical squamous cell carcinoma (case 36) showed high expression of AIB1, in which more than 70% cancer cells showed high staining of AIB1 protein in the nuclei (200×).

Initially, we performed Western blotting to examine the protein levels of AIB1 in three cervical squamous carcinoma cells (SiHa, CaSki and HCC 94). We found that all three cell lines showed higher levels of endogenous AIB1 than that in non-neoplastic cervical benign tissues (Figure 1A). Using the criteria described above, the high expression of AIB1 was observed in 57 of 108 (52.7%) of the cervical cancer and in 9 of 30 (30.0%) of normal cervical tissues (Figure 1B-F). The high expression of AIB1 was significantly correlated with CRT response (*r* = 0.235, *P* = 0.014), clinical stage (*r* = 0.278, *P* = 0.003), T status (*r* = 0.240, *P* = 0.027), N status (*r* = 0.214, *P* = 0.021) and M status (*r* = 0.203, *P* = 0.015) (Table 1). No obvious relativity was found between AIB1 expression and clinicopathologic variables, such as age and WHO grade (*P* > 0.05, Table 1).

Further analysis of the T, N and M groups showed that, the frequency of AIB1 high expression in T3/4, N1 and M1 was especially higher than that in T2, N0 and M0, respectively. This data implied that high expression of AIB1 might contribute to the tumor proliferation, invasion, the metastasis of lymph nodes and distant in cervical cancer.

### Relationship between AIB1 expression and the CRT response

A total of 46 patients achieved CR, in which 18 (39.13%) of them exhibited high expression of AIB1. The rate of CR in AIB1 high expressed group was obviously lower than that in AIB1 low expressed group (39.13% *vs* 60.87%, *P* < 0.05).

### Correlation between AIB1 expression and cervical cancer patient prognosis

**Figure 2:**
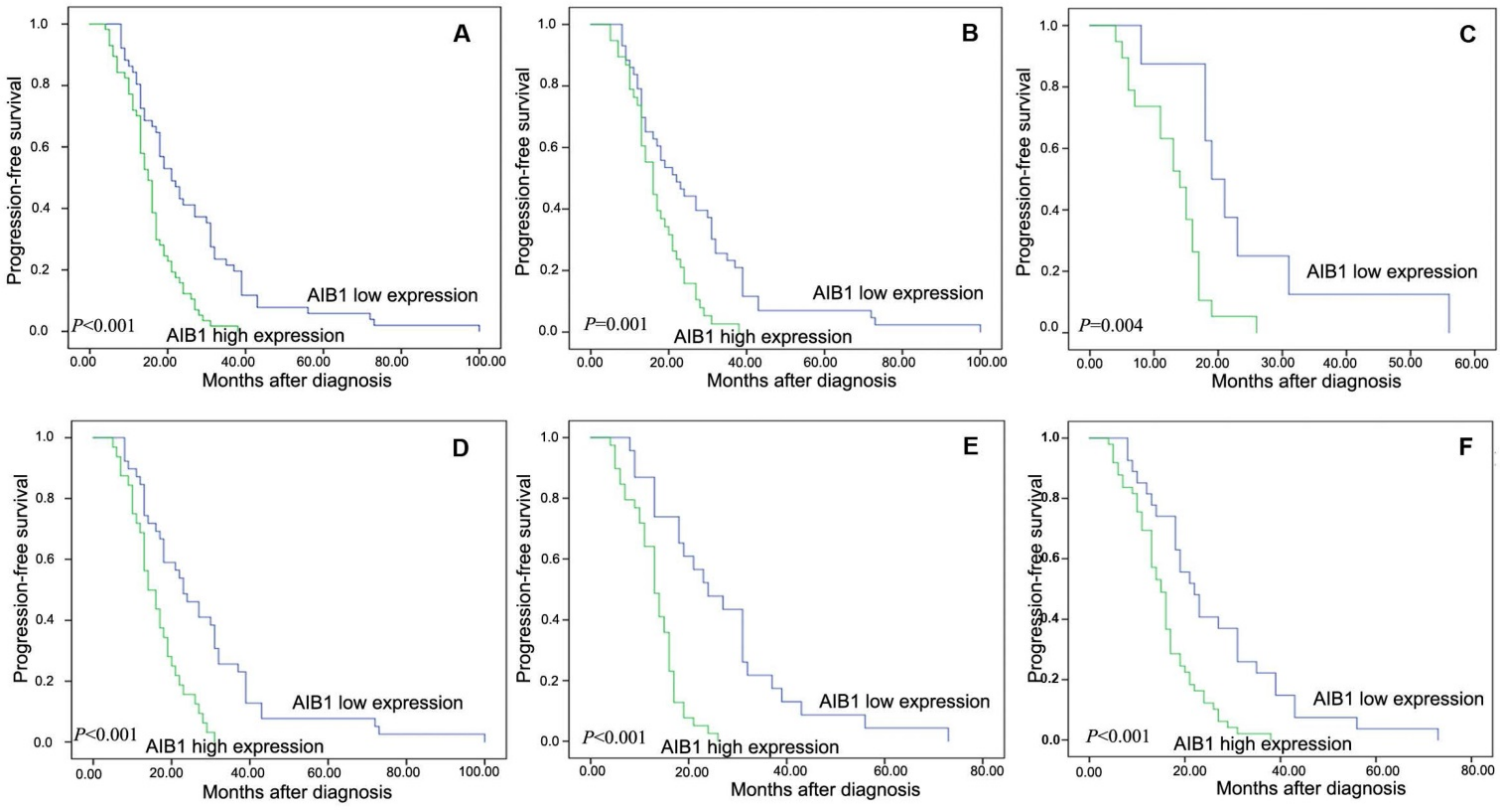
Kaplan-Meier survival analysis of the AIB1 expression in total cohort and different subsets of cervical cancer patients. **A**, Total cohort (high expression =57cases, low expression=51 cases). **B**, M0 subset (high =38 cases, low = 43 cases). **C**, M1 subset (high =19 cases, low = 8 cases). **D**, N0 subset (high =32 cases, low = 39 cases). **E**, non-CR subset (high =39 cases, low = 23 cases). **F**, III+IV subset (high = 49 cases, low = 27 cases). The *P* value was calculated using a log-rank test.

All of the 108 patients with cervical cancer, none was lost to follow-up and the median observation period was 17 months (4-100 months). The average and median numbers of progression-free survival (PFS) in AIB1 high expressed group were 15.7 months and 15.0 months respectively, while the average and median numbers of PFS in AIB1 low expressed group was 26.2 and 21.0 months. Univariate analysis demonstrated a significant impact of certain clinicopathologic prognostic parameters on patient's PFS, such as AIB1 expression (*r* =-0.363, *P < 0.01*), WHO grade, N status, M status and CRT response (*P* <0.05, Table 2). AIB1 high expression was evaluated to correlate closely with poor PFS for the whole cohort and could further stratify patient survival in N0, M0/M1, III+IV stage and non-CR (*P* <0.05, Figure 2A-F). In the N0 and M0 groups, lymph nodes and distant metastasis appeared earlier in AIB1 high-expression group than in AIB1 low-expression group (15.61months vs 26.04 months, average time).

Further COX regression analysis indicated that AIB1 expression and CRT response were independent predictors of PFS (*P* < 0.05, Table 3), with HR is 2.597; 95% CI= 1.667-3.991; *P* < 0.001, and 0.651; 95% CI= 0.439-0.965; *P* = 0.033, respectively (Table 3), whereas, no significant correlation was observed between PFS and other variables, such as N status and M status (*P* > 0.05, Table 3).

### ShRNA-mediated AIB1 knockdown increases chemosensitivity of cervical cancer cells

**Figure 3:**
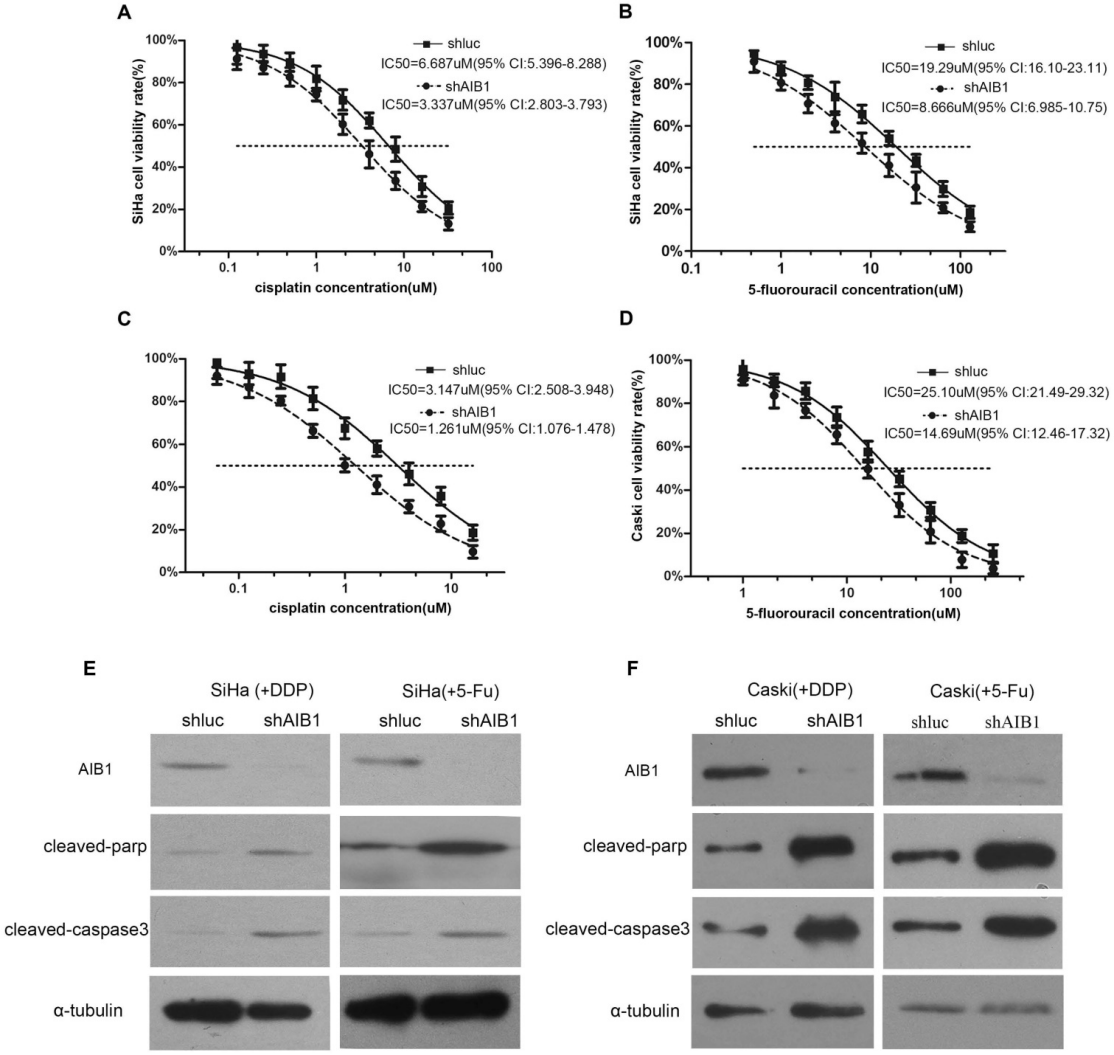
Lentivirus-mediated AIB1 silencing enhances the chemosensitivities of cervical cancer cells. **A**-**D*,*** Dose-response curves of cisplatin or 5-Fu in SiHa and CaSki cells. AIB1-shRNA infected SiHa and CaSki cells showed more sensitive to cisplatin and 5-Fu than parental control cells. IC50 values are shown below. Data are Mean ± SD (n=3, P<0.05). **E** and **F**, After treated cells with cisplatin (IC30) or 5-Fu (IC30) for the 24 hours, the cleaved PARP and cleaved caspase-3 were detected in AIB1-shRNA and Luc-shRNA infected SiHa and CaSki cells by western blot.

Next, we selected SiHa and CaSki cells, the two AIB1 high-expressed squamous cervical cancer cell lines (Figure 1A), to further assess the chemoresistant role of AIB1 in cervical cancer. Since cisplatin and 5-Fluorouracil (5-Fu) are commonly recommended for the cervical cancer treatment, the IC50 values of these two chemotherapeutic agents were examined in SiHa and CaSki cells. As shown in Figure 3A-D, the lentivirus mediated knockdown of AIB1 substantially decreased the IC50 values of cisplatin and 5-Fu in both SiHa and CaSki cells. Consistently, silencing AIB1 also increased cisplatin or 5-Fu-induced levels of cleaved caspase-3 and cleaved PARP in these two cell lines (Figure 3E and 3F). These data indicated that AIB1 play an important role in modulating chemosensitivity in cervical cancer cells.

### Radiosensitivity analysis of human cervical cancer cells by clonogenic cell survival

**Figure 4:**
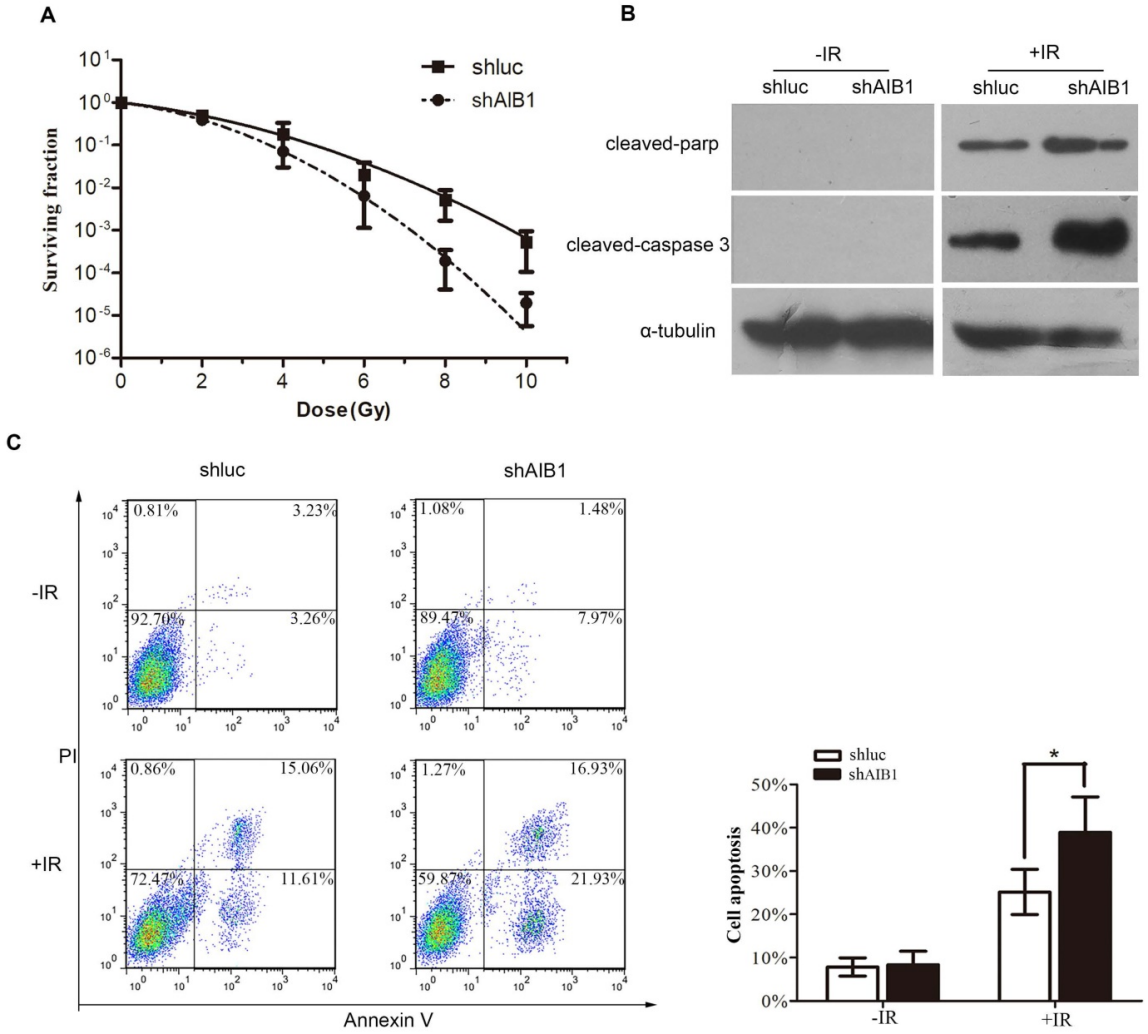
AIB1 depletion increases the sensitivity of cervical cancer cells in response to ionizing radiation. **A**, Clonogenic survival of SiHa-shAIB1 and SiHa-shluc cells. Cells were exposed to increasing doses of radiation as indicated. After 12 days, colonies more than 50 cells were counted and survival curves were fitted according to the linear-quadratic mode. Results were obtained from three independent experiments. All results were from three independent experiments. **B**, After irradiated shAIB1-Siha and shluc-Siha cells with 3Gy or 0 Gy x-ray, the cleaved caspase-3 and cleaved PARP were detected by Western blot analysis. Experiments were performed three times and a representative result is shown. **C**, SiHa-shAIB1 and SiHa-shluc cells were initally treated with or without IR. 36 hours later, the proportion of apoptotic cells were determined by PI/Annexin V assay. Data described are the Means ± SD of triplicates (*P<0.05, Student's t-test).

As our above data indicated that high expression of AIB1 is associated with the poor response to definitive chemoradiotherapy, thus, we performed standard clonogenic formation assay to further determine the anti-radiotherapy effect of AIB1 in cervical cancer cells. The SiHa-shluc and SiHa-shAIB1 cells were irradiated with increasing doses of radiation (0, 2, 4, 6, 8 and 10 Gy), and the survival data were fitted to the linear-quadratic model. The data showed that the clonogenic survival fractions were substantially decreased in SiHa-shAIB1 cells when compared to corresponding control SiHa-shLuc cells (Figure 4A). The radiobiological parameters including the α value, the β value, the α/β value, SF2, and the sensitization enhancement ratio (SER) produced by the LQ model are presented in Table 4. After AIB1 knockdown, the SF2 of SiHa cells was obviously decreased resulting with SER value of 1.264. This result suggested that AIB1 is a radiation-resistant factor in cervical cancer cells.

### Depletion of AIB1 enhances radiation-induced apoptosis in cervical cancer cells

Since apoptosis is a crucial mechanism by which IR exerts its therapeutic response 33 and faulty apoptosis is a known mechanism causing poor response to radiation therapy, in this step, we examine whether silencing AIB1 could enhance IR-induced apoptosis in cervical cancer cells. Using Annexin V/PI staining assay, we noticed that without IR treatment, depletion of AIB1 alone did not induce cell apoptosis in SiHa cells (Figure 4C). However, after cells received 2Gy irradiation, SiHa-shAIB1 cells exhibited a significant increase of apoptotic cells proportion when compared to control SiHa-shluc cells (38.92 ± 8.19 vs. 25.15± 5.23, respectively; *P* < 0.05, Figure 4C).

To further characterize apoptotic activity of AIB1, Western blot analysis was conducted to detect levels of critical apoptotic proteins such as cleavage of caspase-3 and PARP. Consistently, in the absence of IR, no detectable changes of these proteins were observed after AIB1 was silenced. When cells were exposed to 2Gy irradiation, the amount of these proteins was remarkably higher in SiHa-shAIB1 cells than that in SiHa-shluc cells (Figure 4B). These data provided evidence indicating that radiosensitizing effects of AIB1 knockdown were due to enhancing IR-induced cell apoptosis.

## Discussion

AIB1, identified as a coactivator for nuclear steroid hormone receptors, was initially found to be amplified and overexpressed in breast cancer 12, and it has subsequent been shown to be high expressed in other hormone-dependent tumors, such as prostate 34, 35, and ovarian cancer 36, and endometrial carcinoma 37. It had been well documented that AIB1 promotes cancer initiation, proliferation, tumorigenesis, invasion, and metastasis both in breast and prostate cancer, and that high expression of AIB1 was closely associated with an ascending clinical stage and/or poor patient prognosis in these two hormone-sensitive malignancies 15-19. Significantly, several recent studies reported that overexpression of AIB1 enhances the agonist properties of tamoxifen, and dissociation of AIB1 from estrogen receptor (ER) inhibits breast cancer cell growth and consequently restores tamoxifen sensitivity in resistant breast cancer cells 38-40. On the other hand, AIB1 was also identified as a coactivator of a broad spectrum of non-hormonal transcription factors, and facilitates hormone-independent cancers tumorigenesis through multiple hormone-independent signal transduction pathways, including E2F1 signal pathway and NF-κB signal pathway 25, 41, 42. However, the relationship between AIB1 expression and tumor chemoradioresistance was rarely reported.

In this study, we initially examined the expression levels of AIB1 by IHC in 30 normal cervical tissues and in 108 primary cervical cancer samples treated with definitive CRT. Our results showed that the expression of AIB1 in all the normal cervical tissues specimens exhibited absent or at low levels. In contrast, high expressed AIB1 was frequently detected in most of our primary cervical cancer samples. Additionally, the significant correlation between AIB1 expression and clinical stage, T status, N status and M status was observed our chi-square test, which indicated a potential carcinogenic effect of AIB1 in cervical cancer. Similar results were also observed in other hormone-independent cancers, such as gliomas 21, gastric 22, and colorectal carcinomas 24, in which up-regulated expression of AIB1 was found and correlated with an advanced clinical stage and/or poor prognosis.

Our univariate analysis revealed that, AIB1 high expression not only play an important role in the tumorigenic process of cervical cancer (advanced invasion, extra-regional lymph node metastasis and distance metastasis), but also associate closely with an ascending clinical stage and/or poor prognosis of cervical cancer. More importantly, the multivariate Cox regression analysis indicated that AIB1 expression was a significantly independent prognostic factor of cervical cancer patients treated with definitive chemoradiotherapy. Furthermore, AIB1 expression could stratify patient survival in N0, M0/M1, III+IV stage and non-CR (Figure 2B-F). These might provide some evidences in identifying the patients who actually need and might benefit from the multimodality therapies, thus optimize individual therapeutic management.

However, we did not find that high expression of AIB1 was associated with poor overall survival. This might be caused by the reasons that, the good survival of cervical cancer which received the CRT, the 3 years survival rate was >70% 43; our median observation period was 17 months, it was insufficient to observe long-term prognosis in cervical cancer; the OS (overall survival) was only observed in 17 cases of patients (date not show). Longer-term follow-up studies will enable us to capture the impact of AIB1 expression on overall survival of cervical cancer patients. In subsequent studies we will continue observe the survival of these cervical cancer patients, and expect achieve more evidence to support our finding.

To further support this notion, we selected two AIB1 high expressed squamous cervical cancer cell lines (SiHa and CaSki) to establish AIB1 stably silenced cells. The following cell viability and IC50 assay showed that knockdown of AIB1 substantially reduced IC50 value of SiHa and CaSki cells under cisplatin or 5-Fu treatment. Consistently, when AIB1 was silenced, the amount of two important apoptosis-related protein, i.e., cleaved caspase-3 and cleaved PARP, were also obviously increased in both SiHa and CaSki cells which received cisplatin/5-Fu treatment (Figure 3A-F).

As a hormone receptor coactivator, there was accumulative evidence showed that the upregulated expression of AIB1 cause resistance of endocrine therapy in hormone-dependent tumors, especially in tamoxifen resistance of breast cancer.

However, there are only few studies regarding the relationship between AIB1 expression and the resistance of hormone-independent cancers to cytotoxic drugs. By reviewing literature, only Qiang Chen and his colleagues reported that down-regulation of AIB1 enhanced the sensitivity of tumor cells to cisplatin in cholangiocarcinoma 44, which is similar with our present result. Collectively, these results suggest that AIB1 might be not only an inducer of endocrine therapy resistance, but also a predictive marker of tumor resistance to cytotoxic drugs.

Additionally, we further use L-Q model to evaluate the effect of AIB1 on cervical cancer cell radiosensitivity. The SF2 of SiHa cells was obviously reduced when AIB1 was repressed, suggesting that AIB1 might be a radiation-resistant enhancer in cervical cancer. Moreover, the following apoptosis assay indicated that depletion of AIB1 can markedly increase IR-induced cell apoptosis. This result implied that there could be some unknown apoptosis pathways contributed to AIB1's radioresistant effect on cervical cancer cell. Recently, several important studies reported that AIB1 could disturb cell apoptosis via Akt signaling in prostate cancer 34 and head and neck cancer 45, 46. Significantly, the relationship between activation of AKT pathway and tumor radioresistance was also well elucidated in many studied 47-49. Thus, it is conceivable that AIB1 may attenuate radiation-induced cell apoptosis by activation of AKT pathway, resulting with poor response to radiotherapy.

Clearly, more works are needed to further confirm this hypothesis. Nevertheless, this is the first study to report that the high expression of AIB1 in cervical cancer cells contributes to the resistance to IR. Our results not only provide a basis for the concept that high expression of AIB1, as detected by IHC, may be a promising predictor of aggressive cervical cancer patients with poor response to CRT, but also suggest that it might serve as a new potential target to overcome the resistance of radiotherapy in cervical cancer.

## Figures and Tables

**Table 1:** Clinicopathologic correlation of AIB1 expression in cervical cancer.

		AIB1 expression		*P* value ^a^
Variables	All cases	High expression	Low expression	
Age ^b^				0.350
≤50	75	41(39.6)	34(35.4)	
>50	33	16(17.4)	17(15.6)	
WHO grade				0.925
G1	49	25(25.9)	24(23.1)	
G2	38	21(20.1)	17(17.9)	
G3	21	11(11.1)	10(9.9)	
FIGO stage				0.003
II stage	32	9(16.9)	23(15.1)	III vs IV=0.465
III stage	42	25(22.2)	17(19.8)	II vs IV=0.001
IV stage	34	23(17.9)	11(16.1)	II vs III=0.007
T status				0.027
T2	37	13(19.5)	24(17.5)	T3 vs T4=0.065
T3	45	27(23.8)	18(21.3)	T2 vs T4=0.018
T4	26	17(13.7)	9(12.3)	T2 vs T3=0.025
N status				0.021
N0	71	32(37.5)	39(33.5)	
N1	37	25(19.5)	12(17.5)	
M status				0.015
M0	82	38(43.3)	44(38.7)	
M1	26	19(13.7)	7(12.3)	
CRT response				0.014
CR	46	18(24.3)	28(21.7)	
Non-CR	62	39(32.7)	23(29.3)	

^a^ Chi-square test; ^b^ Median age; T status: T stages; N status: lymph nodes status; M status: Distant metastasis; CR: Complete response/complete remission; CRT: chemoradiotherapy; *P*: *P* value;

**Table 2:** Univariate survival analysis.

		FPS (progression-free survival)		*P* value ^a^
Variables	All cases	Median (SE)	95% CI	
Expression				0.000
High	57	15(1.029)	12.982~17.018	
Low	51	21(2.550)	16.002~25.998	
Age ^b^				0.064
≤50	75	18(1.176)	15.695~20.305	
>50	33	14(1.595)	10.874~17.126	
WHO grade				0.030
G1	49	20(1.500)	17.061~22.939	
G2	38	14(1.761)	10.548~17.452	
G3	21	13(1.907)	9.262~16.738	
T status				0.161
T2	37	17(2.021)	13.039~20.961	
T3	45	16(1.341)	13.371~18.629	
T4	26	17(1.530)	14.002~19.998	
N				0.032
N0	71	18(1.684)	14.700~21.300	
N1	37	16(0.995)	14.049~17.951	
M				0.031
M0	82	18(1.727)	14.614~21.386	
M1	26	16(1.291)	13.470~18.530	
FIGO stage				0.239
II	32	17(2.258)	12.574~21.426	
III	42	16(1.573)	12.917~19.083	
IV	34	17(1.200)	14.648~19.352	
CRT response				0.047
CR	46	20(1.884)	16.307~23.693	
Non-CR	62	15(1.181)	12.685~17.315	

^a^ Log-rank test; ^b^ Median age; CI: confidence interval; *P*: *P* value; T status: T stages; N status: lymph nodes status; M status: Distant metastasis; CR: Complete response/ complete remission; CRT: chemoradiotherapy;

**Table 3:** Multivariate Cox regression analysis.

	B	SE	Exp(B)	95% Exp(B) CI		*P*
AIB1 expression	0.948	0.223	2.579	1.667	3.991	0.000
CRT response	-0.429	0.201	0.651	0.439	0.965	0.033

B: beta; SE: Exp(B): The exponent of B; CI: confidence interval; *P*: *P* value;

**Table 4:** Comparison of radiobiological parameters of SiHa cells infected with sh-luc or sh-AIB1.

	α value (Gy^-1^)	β value (Gy^-2^)	α/β value (Gy)	SF2	SER
SiHa-shluc	0.256	0.048	5.352	0.495	1.264
SiHa-shAIB1	0.276	0.097	2.853	0.392	

SF2: survival fraction at 2Gy; SER: sensitizing enhancement ratio.
